# Purification and characterization of recombinant human translation initiation factor eIF3

**DOI:** 10.1002/pro.70388

**Published:** 2025-12-23

**Authors:** Irene Diaz‐Lopez, Yuliya Gordiyenko, Philipp K. Zuber, Xueyan Li, V. Ramakrishnan

**Affiliations:** ^1^ MRC Laboratory of Molecular Biology Cambridge Biomedical Campus Cambridge UK

**Keywords:** eIF3, high yield, multifactor complex purification, translation initiation

## Abstract

Eukaryotic translation initiation factor 3 (eIF3) is an essential factor in protein synthesis. In mammals, it is a ~800 kDa complex composed of 12–13 subunits. Biochemical and mechanistic studies of the function of eIF3 and its individual subunits in translation require purified eIF3. However, current strategies for obtaining mammalian eIF3 rely on purification of the endogenous factor from cultured human cells or rabbit reticulocytes lysates, both of which are expensive and time consuming. Here we present a recombinant insect‐cell expression and purification system for human eIF3 which allows the purification of large amounts of functional, homogeneous eIF3 efficiently and cost‐effective, while also enabling engineering, such as generation of site‐specific mutations in the factor for functional studies.

## 1 INTRODUCTION

Protein synthesis, or translation, is one of the key steps in gene expression. Translation is divided into four steps, initiation, elongation, termination, and recycling. It has become clear over the past decade that the initiation phase is a key step in the regulation of translation. Generally, initiation starts with the small ribosomal subunit and free mRNA and ends with the mRNA start codon and a special initiator tRNA positioned in the P site of the whole ribosome. While initiation occurs in all kingdoms of life, the process is far more complex in eukaryotic cells than in prokaryotes. In addition to the small subunit and mRNA, several protein initiation factors are required for the process. In bacteria there are only three initiation factors, while in eukaryotes there are about 10 initiation factors, significantly increasing the complexity of the eukaryotic system (Brito Querido, Díaz‐López, & Ramakrishnan, 2024).

Eukaryotic translation initiation factor 3 (eIF3) is the largest translation initiation factor and is involved in every step of translation initiation. In mammals it comprises 12 subunits (eIF3a–eIF3i and eIF3k–eIF3m) and an additional loosely associated subunit (eIF3j). Due to its large size, it spans the entire initiation complex, interacting with the small ribosomal subunit (40S in eukaryotes), the mRNA, and every other initiation factor, except eIF5B (Brito Querido, Díaz‐López, & Ramakrishnan, 2024). It regulates virtually every aspect of initiation from recruitment of the 40S to mRNA, to start codon recognition.

The eIF3 subunits eIF3a, eIF3c, eIF3e, eIF3f, eIF3h, eIF3k, eIF3l, and eIF3m form an octameric core, which together with eIF3d binds to the small ribosomal subunit (40S) near the exit of the mRNA channel. Those subunits also interact directly with the 40S, the ternary complex (TC, composed of eIF2, Met‐tRNA_i_, and GTP), eIF1, eIF1A, and eIF5, forming the 43S pre‐initiation complex (PIC) during the first steps of initiation. During the mRNA recruitment and AUG recognition, the eIF3 core subunits interact as well with the mRNA and the cap‐binding complex eIF4F, together forming the 48S complex (Brito Querido, Díaz‐López, & Ramakrishnan, 2024). All those interactions are crucial for the recruitment of the ribosome to the mRNA at the beginning of translation initiation, yet, the function of many of the eIF3 core subunits are still not fully understood (des Georges et al., 2015; Masutani et al., 2007).

The subunits eIF3b, eIF3g, and eIF3i form an independent module within the eIF3 complex. Until recognition of the initiation codon, they bind to the 40S near the entry of the mRNA channel, interacting with the mRNA and the RNA helicase eIF4A, thus likely contributing to the regulation of 48S scanning though the mRNA 5′ untranslated region (5′UTR) (Valášek et al., 2017). eIF3g interacts directly with the 40S and is the closest to the mRNA entry channel. Therefore, it has been proposed to act as an important co‐factor for the function of proteins that regulate translation initiation at the mRNA entry channel, such as Pdcd4, a human tumor suppressor that inhibits translation initiation under specific conditions as nutrient deprivation (Brito Querido, Sokabe, Díaz‐López, Gordiyenko, Zuber, et al., 2024), or SARS‐CoV 2 Nsp1, that binds to the mRNA channel and inhibits host translation initiation to overtake the translational machinery (Abaeva et al., 2023).

The regulation of eIF3 expression plays an important role in many cellular processes such as activation of T‐regulatory cells (Volta et al., 2021) and is implicated in translation of viral proteins upon infection (Neupane et al., 2020). Specific individual eIF3 subunits are dysregulated in numerous human cancers, and their overexpression in cells can result in malignant transformation (Hershey, 2015).

Biochemical and structural studies on eIF3 currently rely on purification of the endogenous factor from human cells (HEK, HeLa) or rabbit reticulocyte lysate (Damoc et al., 2007; Pisarev et al., 2007). These methods are time‐consuming and expensive. The large number of subunits, many of which interact intimately in the complex also means that overexpression will require the simultaneous expression of multiple subunits for proper folding. Previous attempts to express the eIF3 complex in *Escherichia coli* (Sun et al., 2011), though partially successful, involve separate expression of several subunits, and required deletion of large portions of eIF3a and eIF3c to obtain a stable construct. Bacterial expression also omits post translation modifications, which are very likely important for eIF3 function and stability (Andaya et al., 2014; Damoc et al., 2007).

Here, we present an efficient system for purification of functional full‐length human eIF3 from overexpression in insect cells. This method not only provides a significantly higher final yield than purification of the endogenous factor but also makes the engineering of eIF3 possible. This includes introduction of mutations or tags, deletion of parts of, or entire subunits, or fluorescent labelling at specific sites in defined subunits, which in turn allows detailed functional studies on eIF3.

## 2 RESULTS

### 2.1 Construction of recombinant plasmids and bacmid generation

To enable simultaneous overexpression of human eIF3 subunits in insect cells, we used the biGBac strategy. This system has been shown to allow the co‐expression of multifactor complexes containing up to 20 subunits (Weissmann et al., 2016).

**FIGURE 1:**
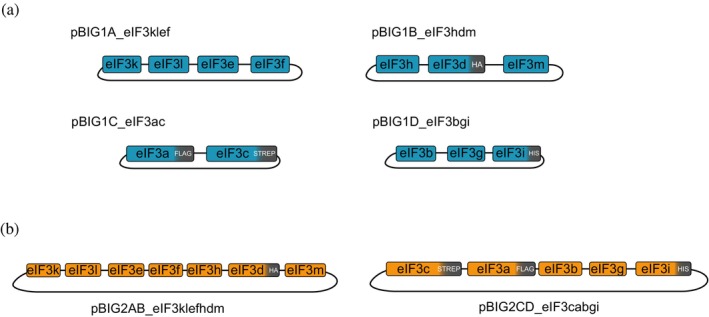
Schematic representation of the eIF3 subunits organization in the plasmids. Arrangement of eIF3 subunits in the four pBIG1 plasmids (a) or two pBIG2 plasmids (b). Coding sequences of the subunits are represented as blue/orange boxes respectively, and tag sequences are represented in gray boxes.

We first obtained synthetic coding sequences of the 13 human eIF3 subunits, codon optimized for expression in insect cells, cloned into pACEBac1. Different tags were added to the eIF3a, eIF3c, eIF3d, and eIF3i subunits (Figure S1, Supporting Information), that allow detection by Western blot or specific purification of the fully formed complex by affinity chromatography. The entire insect cell expression cassettes of all eIF3 subunits, except for eIF3j, were then combined into the different pBIG1 vectors (Figure 1a). We rationalized the organization of the subunits based on the known structure of the eIF3 complex (core composed of eIF3a, eIF3c, eIF3e, eIF3f, eIF3h, eIF3k, eIF3l, and eIF3m), eIF3d that binds to the core, and the peripheral eIF3bgi complex. Since the eIF3j subunit is only loosely associated with the complex, we decided to not co‐express it with the other subunits. Instead, eIF3j can be easily expressed and purified separately and then added to the 12‐subint eIF3 complex, if required (des Georges et al., 2015). To generate the four pBIG1 plasmids, gene cassettes of the eIF3 subunits were amplified by PCR (Figure S2a) and subcloned into the pBIG1 vectors. We verified the correct assembly of eIF3 subunits in the pBIG1 plasmids by enzymatic digestion (Figure S2b) and DNA sequencing. The resulting plasmids can be used for bacmid generation and separate expression of the different groups of eIF3 subunits.

The subunits were then combined in two different pBIG2 plasmids (Figure 1b) by enzymatic digestion and Gibson assembly, following the protocol described for pBIG2 assembly (Weissmann et al., 2016). We verified the correct assembly of the plasmid by digestion and DNA sequencing (Figure S2c).

The pBIG2 plasmids were transformed into DH10Bac cells harboring a bacmid with a YFP reporter (made in house) following standard protocols. After 2 days of incubation at 37°C, colonies showing successful integration of the expression cassettes into the bacmid were selected and amplified the bacmid to transfect insect cells.

### 2.2 Baculovirus preparation and eIF3 expression

**FIGURE 2:**
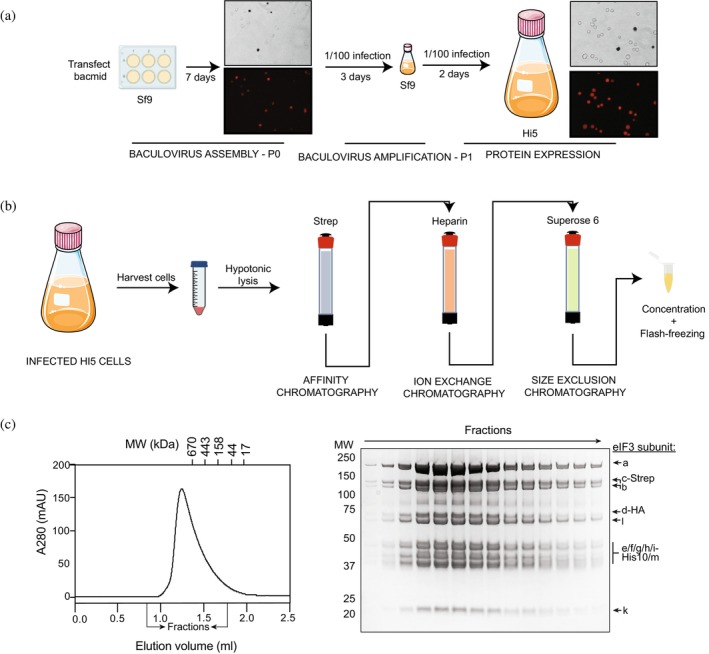
Schematic representation of eIF3 complex expression and purification protocol. (a) Generation of baculovirus for eIF3 expression. Sf9 cells are used to produce P0 and P1 viral progenies. Cell viability was assessed by trypan blue staining and bright field microscopy (top panels, dark cells correspond to death cells). Infected cells were identified based on YFP expression by fluorescence microscopy (red cells are infected cells). (b) Main protocol for purification of eIF3 complexes from over‐expressed in Hi5 insect cells. (c) Analysis of the integrity of purified eIF3 complex by analytical SEC. Chromatogram of SEC run using a Superose6 3.2/300 column of eIF3 expressed in insect cells and purified. Coomassie‐stained SDS polyacrylamide of the fractions indicate in the chromatogram.

Bacmids were transfected info Sf9 insect cells to generate the first viral progeny (P0). We collected the P0 5 days post transfection (dpt) and used it to subsequently infect Sf9 cells for amplification of the progeny (yielding P1). Three days post infection (dpi) the virus was collected. P1 was used to infect insect cells for protein expression (Figure 2a). For co‐infection, the P1 viruses of different constructs were mixed in a 1:1 (volume:volume) ratio and cells were co‐infected at the indicated ratios P1‐mix:cells.

To optimize expression conditions, we varied parameters such as insect cell line, virus:virus ratio of infection, and expression time. Lysates from small‐scale expression tests were then subjected to streptavidin affinity chromatography to assay expression and assembly of the eIF3 complex assembly (Figure S3a,b). Based on protein levels and presence of all the eIF3 factors in the complex, the optimal conditions for eIF3 expression were found to use the Hi5 cell line, a volumetric 1:1 P1 viral ratio and 48 h of protein expression (over 95% cell viability). We observed the enrichment of an eIF3acb subcomplex, indicating that further purification to obtain a stochiometric complex is needed.

### 2.3 Purification of recombinant eIF3

To purify overexpressed eIF3 in large scale, insect cells obtained 2 dpi were lysed in hypotonic lysis buffer to prevent proteolytic degradation as described in material and methods. The resulting raw extract was subjected to three different types of column chromatography to ensure isolation of the full complex (Figure 2b).

First, the cell extract was applied to a streptavidin affinity column to enrich the eIF3 complex via the Strep tag on eIF3c. After elution with biotin, the peak fractions were analyzed by SDS‐PAGE. All 12 eIF3 subunits were present in the analyzed fractions (Figure S4a). As observed before, the subunits in the resulting sample were not stochiometric, indicating that further purification is required to obtain a homogenous complex. Nucleic acid contamination was observed based on the A260/A280 ratio. This was expected as eIF3 is known to bind RNA (REF). To remove nucleic acid contamination, the proteins eluted from the affinity column were thus subsequently loaded onto a Heparin column, which also removes any possible nucleic acid contamination. After elution via a 100–400 mM KCl gradient, the peak fractions were analyzed by SDS‐PAGE (Figure S4b). After the Heparin column, different eIF3 subcomplexes were still present. The fractions containing all eIF3 subunits and showing a low A_260_/A_280_ ratio (indicative of the absence of nucleic acids) were collected, concentrated, and buffer exchanged to a final concentration of 150 mM KCl to ensure the integrity of the complex. To ensure a homogenous and stochiometric complex, the combined fractions were finally applied to a Superose 6 size exclusion chromatography (SEC) column and the fractions were analyzed by SDS‐PAGE (Figure S4c). Fractions with the correct stoichiometry of the factors and a good A260/280 ratio were pooled and concentrated for further analysis. We obtained 1 mg of protein from 15 g of total wet cell pellet (obtained from 1 L cell culture). Final purified complex was analyzed by size exclusion chromatography after two rounds of freeze and thaw cycles to ensure stability of the complex (Figure 2c).

**FIGURE 3:**
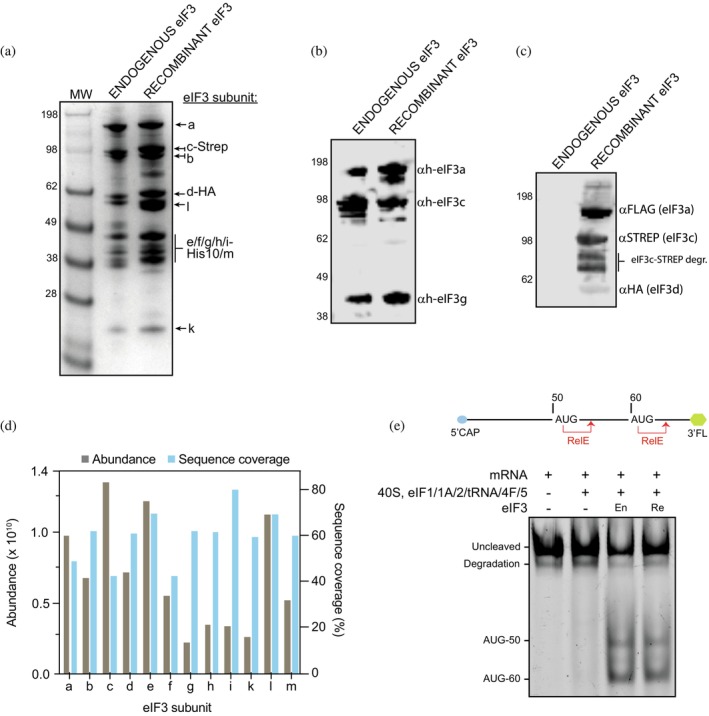
Analysis of recombinant eIF3 complex purified from insect cells. (a) Coomassie‐stained SDS polyacrylamide gel of native eIF3 and recombinant purified eIF3; 7 pmoles of each complex were loaded per lane. All the eIF3 subunits are present in the recombinant purified complex in an equimolar ratio. (b) Western blot analysis of endogenous and recombinant purified eIF3 complexes. Antibodies recognizing human eIF3 subunits a, c and g were used. 3 pmoles of each complex were loaded per lane. All the eIF3 subunits analyzed were present in both complexes. (c) Western blot analysis of endogenous and recombinant eIF3 complex (3 pmoles of each complex were loaded in each lane). Antibodies recognizing the tags present in the recombinant eIF3 complex were used (FLAG, STREP, and HA). All tags were present in the recombinant eIF3 complex but not in the endogenous complex. (d) Mass spectrometry analysis of purified eIF3 recombinant complex. The plot shows the abundance and sequence coverage of the 12 eIF3 subunits. (e) RelE assay to assess the activity of the eIF3 complex. Scheme of the mRNA used is shown, with two AUG codons positioned at 50 and 60 nt from the CAP. The mRNA is labeled at the 3′ end with fluorescein for detection. Without eIF3, the initiation complex cannot recognize any AUG. The addition of endogenous (En) or recombinant (Re) eIF3 complex promotes AUG recognition in comparable levels (1 replicate).

To assay the integrity of the complex after purification, we compared the recombinant eIF3 purified with endogenous eIF3 purified from HeLa cells (Figure 3). The same amount of endogenous and recombinant eIF3 complex was analyzed by SDS‐PAGE and Coomassie‐staining. All 12 eIF3 subunits could be identified in the recombinant complex, in quantities and apparent molecular weight identical to those of the endogenous control (Figure 3b). Western blotting against human eIF3a, eIF3c, and eIF3g of the recombinant and endogenous eIF3 complexes showed that eIF3 subunits were present in the recombinant complex to the same extent as in the endogenous complex (Figure 3c, left panel). To further ensure that the eIF3 complex contained the overexpressed subunits, a Western blot analysis showed the FLAG, Strep and HA tags introduced recombinantly in the eIF3 complex were present in the insect cell expressed complex but not in the endogenous complex (Figure 3c, right panel).

Finally, mass‐spectrometry analysis confirmed the presence of all 12 human eIF3 subunits in the recombinant complex and the absence of mayor contaminants (Figure 3d and Table S1). Together, those results highlight the purity and integrity of the obtained recombinant eIF3 human complex.

### 2.4 Activity of recombinantly produced eIF3

To assess the activity of the recombinant human eIF3 complex, we used a reconstituted human translation initiation system to assemble 48S complexes in vitro. Complex formation was then visualized by a RelE cleavage assay (Querido et al., 2020). The endonuclease RelE specifically cleaves mRNA in the empty A site of the complete ribosome or its small subunit. If initiation complexes are assembled in the absence of the large ribosomal subunit and elongation factors, initiation will stall at the start codon recognition step, resulting in an accumulation of 48S complexes with the AUG in the P site and an empty A site. This complex constitutes a substrate for cleavage with RelE, resulting in a defined fragment of mRNA with a size that depends on the position of the AUG codon (Andreev et al., 2008).

To enable detection of RelE cleavage, the test mRNA was labeled at the 3′ end with a fluorophore and the resulting mRNA fragments were analyzed by urea PAGE (Figure 3e). We employed an mRNA used in previous studies that contains AUGs at position +50 and +60 (Querido et al., 2020).

In the absence of an eIF3 complex (but presence of all other factors and components) there was no mRNA cleavage. This is expected since eIF3 is required for initiation. Upon addition of the endogenous eIF3 complex, fragments resulting from initiation complex formation at both AUGs (at positions 50 and 60) could be observed. In the presence of the same concentration of recombinant eIF3 complex, the same efficiency of initiation complex formation was observed. This indicates that recombinant eIF3 complex is active to the same extent as the endogenous complex.

### 2.5 Versatility of the system

**FIGURE 4:**
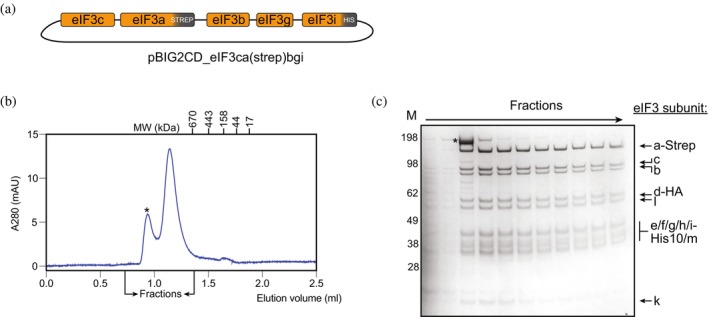
Analysis of purification of eIF3 via a STREP tag on eIF3a. (a) Schematic organization of the pBIG2CD plasmid after genetic modification. The STREP tag in the eIF3c subunit on the original bacmid has been removed. The FLAG tag in the eIF3a subunit in the original plasmid has been replaced by a STREP tag. (b) Chromatogram of analytical SEC run using a Superose6 3.2/300 column of eIF3 complex purified from the eIF3a‐STREP‐tagged modified system (column volume 2.5 mL). The asterisk marks the exclusion (void) volume. SEC sample contains 1 μM eIF3 in 20 μL. (c) Coomassie‐stained SDS‐PAGE gel of the selected fractions. The asterisk marks a contaminant.

To test the robustness of our recombinant human eIF3 complex expression and purification system, we removed the STREP tag from the eIF3c subunit and substituted the FLAG tag on eIF3a for the STREP tag (Figure 4a). Both modifications were introduced via PCR in the pACEBac plasmids encoding the isolated subunits, and the modified subunits were then assembled into pBIG1/2 vectors as before. Following the same workflows for expression and purification, we were able to recover a full stochiometric eIF3 complex (Figure 4b,c). This result indicates that the system is robust and amenable to genetic modifications and suggests that it will be possible to easily introduce mutations (point mutations, insertions, or deletions in any factor), and add different tags and labels for biochemical characterization (such as ones used for single‐molecule experiments), opening the possibility of more sophisticated experiments on the role of eIF3 in translation initiation.

**FIGURE 5:**
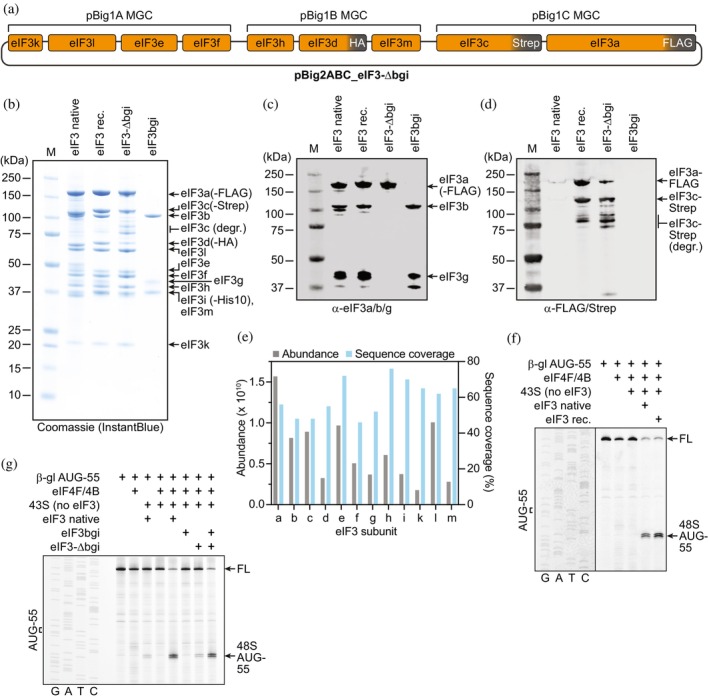
Preparation of eIF3‐∆bgi/eIF3bgi as isolated subcomplexes or in combination as an alternative strategy for preparation of complete eIF3. (a) Schematic organization of the pBIG2ABC_eIF3‐∆bgi plasmid, obtained by combining the pBIG1A/1B/1C multi‐gene cassettes (MGCs; compare Figure 1a). (b) Coomassie‐stained SDS polyacrylamide gel of native eIF3, recombinant eIF3‐∆bgi and eIF3bgi, as well as recombinant eIF3 generated by combining cells from individual expression of the two subcomplexes (5 pmoles of each complex were loaded per lane). Assignment of the bands to subunits (indicated on right) was based on predicted molecular weight and combinatorial analysis of the subcomplexes. (c, d). Western blots against human eIF3a/c/g (C) or FLAG/Strep tag II (D) of the identical samples as in (B); 2 pmoles per (sub‐)complex were loaded. (e) Mass spectrometry analysis of full‐length eIF3 obtained from the pellet‐mixing strategy. The plot shows the abundance and sequence coverage of the 12 eIF3 subunits. (f, g) Toe‐printing assays using an mRNA with beta‐globin like 5′ UTR and a start codon at position 55 (indicated next to the sequencing ladder on the left). The position of the 48S complexes halted at the start codon is indicated on the right construct. Altogether, this indicates that the eIF3 complex obtained using this construct and strategy is functional, homogeneous and contains all subunits in stochiometric amounts.

To further explore the versatility of our recombinant expression system, we sought to make use of the modular organization of eIF3 in a core (comprising subunits eIF3a, eIF3c, eIF3d, eIF3e, eIF3f, eIF3h, eIF3k, eIF3l, and eIF3m) and the eIF3bgi subcomplex, linked through the C‐terminus of eIF3a (Querido et al., 2020; Valášek et al., 2017). The eIF3bgi complex functions as a separate module within the eIF3 complex, that resides on the entry site of the 40S subunit within the 43S PIC/48S complex (Querido et al., 2020) and plays important roles during translation initiation, for example, by providing a platform for positioning eIF4A and 4B during mRNA loading and/or scanning (Brito Querido, Sokabe, Díaz‐López, Gordiyenko, Fraser, & Ramakrishnan, 2024), as well as for the RNA helicase DHX29 during scanning of structured 5′ UTRs (Hashem et al., 2013). The eIF3 core is mainly bound to the solvent side of the 40S subunit and contains the primary binding site for eIF4F (Querido et al., 2020). Uncoupling the functions of the eIF3 core and eIFbgi modules by generating isolated subcomplexes would thus allow characterization of the interactions with their corresponding binding partners and detailed studies of the role of the ribosomal entry site‐localized eIF3 subunits in translation initiation. Finally, combining the two subcomplexes may also represent an alternative strategy for preparation of full‐length eIF3. We therefore generated an expression plasmid for the eIF3 core, henceforth referred to as eIF3‐∆bgi, by combining the pBIG1A–C (Figure 5a) multi‐gene cassettes in pBIG2ABC (Figure 5a). The pBIG1D plasmid encoding eIF3bgi (Figure 5a) was used directly for bacmid preparation. Viruses were prepared individually for both constructs, identical to the pBig2AB/CD strategy. A lysis test of the cell pellets obtained from the P1 virus generation showed that both eIF3‐∆bgi and eIF3bgi express well in isolation and are soluble independent of the presence of one another (Figure S4a). We therefore proceeded to express eIF3‐∆bgi and eIF3bgi separately in Hi5 cells.

We first asked whether the complete eIF3 complex can be obtained by combining pellets from individual expressions of eIF3‐∆bgi and eIF3bgi. We therefore mixed the corresponding cell pellets at an ~1.5:1 (eIF3‐∆bgi:eIF3bgi) mass ratio. Since the expression level of eIF3bgi is considerably higher compared to the subunits of the other subcomplex (Figure S5a), this results in a vast excess of eIF3bgi over eIF3‐∆bgi, ensuring that all binding sites on eIF3a are being saturated. We then isolated eIF3 using the strategy presented above for the other complete recombinant eIF3 complexes, that is, affinity chromatography via the STREP tag on eIF3c, Heparin chromatography and SEC. Overall, we obtained a yield of ~10 mg protein from a combined ~50 g of total wet cell weight (obtained from combined 3 L of culture).

Analysis of the resulting sample by SDS‐PAGE (Figures 5b and S5b), Western blotting (Figure 5c,d), and mass spectrometry (Figure 5e) showed that all eIF3 subunits were present, and that the eIF3 complex contained eIF3bgi at the same stoichiometry as native eIF3. This indicates complete complex formation within the mixed cell lysate, that is, all binding sites on eIF3a for eIF3bgi were covered. Furthermore, the obtained eIF3 complex was homogeneous at 10 μM concentration, as judged by analytical SEC (Figure S5c,d), and had the same molecular weight as native eIF3, as evident from mass photometry (at 50 nM; Figure S4e). Finally, we assessed the functionality of the obtained construct by a ribosomal toe‐printing assay (Figure 5f). This assay is similar to the RelE cleavage assay in that the components to be tested (40S ribosomal subunit, eIFs and initiator tRNA) are allowed to form initiation complexes on an mRNA, usually accumulating as 48S complexes stalled at a start codon. As a read‐out of initiation complex formation, a fluorescently labeled primer is annealed downstream to the site of interest and extended using a reverse transcriptase (RT). Ribosomal complexes stalled on the mRNA will generate a block for RT read‐through (~14–15 nts downstream of the corresponding P‐site codon), which can then be visualized as an additional band on a sequencing gel. The toe‐printing assay confirmed that a 43S PIC lacking eIF3 is unable to support initiation on an mRNA with a 55 nts 5′ UTR (Figure 5f). Using native eIF3 to assemble the 43S PIC resulted in a strong toe‐printing band, corresponding to a 48S complex stalled at the AUG codon at position 55. Compared to native eIF3, we obtained a comparable activity with the recombinant eIF3 purified by this strategy. Altogether, this indicates that functional, homogeneous and complete eIF3 can be obtained in high quantities by combining individually expressed eIF3‐∆bgi and eIF3bgi subcomplexes.

Considering that eIF3‐∆bgi and eIF3bgi are soluble in isolation (Figure S5a) we next sought to obtain the isolated eIF3 modules—a prerequisite for characterization of their roles in translation initiation in future studies. We therefore purified the two subcomplexes independently by affinity chromatography (Streptactin chromatography for eIF3‐∆bgi via the Strep tag on eIF3c, and immobilized metal affinity chromatography for eIF3bgi via a His10 tag on eIF3i), followed by Heparin chromatography and SEC using a Superose6 column, respectively.

The resulting eIF3bgi complex contained the three subunits in stochiometric amounts, as indicated by SDS‐PAGE (Figure 5b,c) and the correct molecular weight in a mass photometry analysis (Figure S5c). Analytical SEC using a Superose6 column showed that the complex was highly homogenous and stable (Figure S5a,b). For eIF3‐∆bgi, we observed some proteolytic degradation during the purification, especially on eIF3c, as evident from additional bands between eIF3d and eIF3c in an SDS‐PAGE gel (Figure 5b) and Western blotting for the Strep tag on eIF3c (Figure 5d). Mass photometry analysis (50 nM protein) showed a main species of the expected mass, but with a small shoulder on the lower molecular weight side indicating degradation (Figure S5d). At higher protein concentrations used for analytical SEC (10 μM), the complex tended to multimerize (Figure S5a,b); however, this effect was reversible, since at lower concentrations (1.5 μM) or higher ionic strength (400 mM instead of 150 mM KCl), the “monomer” peak was significantly higher (Figure S5e).

To assess whether the two subcomplexes are sufficient to promote initiation complex formation, we assessed their activity in a toe‐printing assay (Figure 5g). When added to a 43S PIC lacking eIF3, neither the eIF3bgi nor the eIF3‐∆bgi subcomplex alone allowed substantial initiation complex formation, with no, or only a weak 48S toe‐printing signal, respectively. However, when combining eIF3‐∆bgi and eIF3bgi and allowing a brief incubation at 30°C, the resulting level of initiation complex formation is comparable to that obtained from 43S PIC formed with native eIF3. This suggests that the two isolated subcomplexes can be combined a posteriori into a functional full‐length eIF3 complex. Indeed, both analysis by mass photometry (Figure S5d) and analytical SEC (Figure S5a,b) confirmed that eIF3‐∆bgi associated with the eIF3bgi subcomplex when added together. At the same time, the amount of multimerized eIF3‐∆bgi within the sample strongly decreased (Figure S5a,b), further supporting the notion that eIF3‐∆bgi self‐association can be reversed by dilution or the presence of a suitable binding partner.

Together, these results suggest that (i), a functional eIF3 complex can be reconstituted from isolated subcomplexes and (ii), the eIF3bgi submodule is crucial for efficient translation initiation, either for recruitment of the mRNA and/or subsequent scanning to the start codon, thus setting the stage for future investigations of the dynamics around the factors targeting the ribosomal entry site.

Finally, we used the same approach for the recombinant expression of human eIF2 complex in insect cells. eIF2 is another multicomplex translation initiation factor composed of three subunits, eIF2α, eIF2β, and eIF2γ. We combined the three subunits in one bacmid to generate the baculovirus and infect insect cells following a similar approach described in this paper (Figure S6). The plasmids are available at Addgene. This factor could be purified following a similar protocol described here for eIF3 (data not shown) and opens the possibility to assemble a translation initiation complex with fully recombinant translation initiation factors.

## 3 DISCUSSION

Detailed and controlled in vitro studies of translation initiation require reliable methods to purify all the involved factors. The multi‐subunit translation initiation factor eIF3 is one of the most challenging mammalian initiation factors to purify. Traditional eIF3 purification strategies rely on isolating endogenous complexes from rat and mouse liver, rabbit reticulocyte lysates (RRL) or human cells in culture (Benne et al., 1979; Falvey & Staehelin, 1970; Pestova et al., 1996). As is often associated with isolating proteins from their native sources, these methods suffer from the (low) endogenous expression levels of the target proteins(s), which needs to be compensated by a vast increase in starting materials. This is especially costly for isolation of the human factor, which requires cells from 40 to 60 L of HEK/HeLa culture (Sokabe & Fraser, 2014). Additionally, due to the lack of a tag, the preparations are time consuming, as many purification steps are needed to remove contaminants.

In this study we successfully expressed and purified the 12‐subunit human eIF3 complex in insect cells. Importantly, our recombinant eIF3 is homogenous, stable and shows an activity that is comparable or even better than that of the native complex. Overexpression of recombinant eIF3 complex combines the advantages of obtaining higher yield at a fraction of the cost of obtaining starting material for purification of the endogenous complex, and the possibility of introducing modifications to the system. Most other eukaryotic translation initiation factors are purified following this strategy for in vitro studies (Querido et al., 2020).

Overexpression and purification of eIF3 has been tried before. First attempts of insect cell expression of eIF3 were done expressing the subunits separately (Fraser et al., 2004), or using co‐expression from three different constructs (Masutani et al., 2007), but did result in a non‐stable eIF3 complex with low yield of purification. Overexpression of eIF3 subunits in bacteria, also did not result in a stable complex, probably because of the lack of post‐translational modifications (Sun et al., 2011). More recently, the strategy to introduce a tag on one specific eIF3 subunit and overexpress it in human cells to purify the eIF3 complex has been used (Abaeva et al., 2023). This strategy allows introduction of modifications on the expressed subunit, indicating how important it is to have a robust purification method for eIF3 complex that allows easy modification of the complex.

The eIF3 purification method presented here combines high yield of pure, stable and functional complex with the capability to easily introduce (simultaneous) modification(s) on any eIF3 subunit(s). This approach is faster, less laborious, more straightforward, and cheaper than current purification protocols of endogenous eIF3. The recombinant system allows us to introduce genetic mutations, as well as labels and tags on eIF3 as needed, thus allowing more sophisticated functional studies involving the factor. We consider this method of particular interest to those researchers studying eukaryotic translation initiation.

## 4 MATERIALS AND METHODS

### 4.1 eIF3 individual subunits plasmids

Protein sequences for all human eIF3 subunits were obtained from the UniProt database (Figure S1). For the tags, standard sequences were used and added in frame with the coding sequence of the respective subunits, all of them at the C‐terminus (Figure S1). Additionally, a 3C protease cleavage site was inserted between eIF3c and STREP‐tag, and a TEV protease cleaving site between eIF3a and the FLAG tag.

Synthesis of the eIF3 genes and cloning into plasmids was conducted by Epoch Life Science. All coding sequences were codon‐optimized for human and insect cells' protein expression. A 5′ leader sequence was included, that contained a *Bam*HI restriction site for further cloning and an optimal Kozak sequence immediately upstream of the initiation codon for eukaryotic protein expression optimization. The final sequences were cloned separately into pACEBac1. This vector encodes the entire cassette for insect cell expression; therefore, bacmids can be generated for each individual subunit and expressed in insect cells independently. Sequences and plasmids are available at Addgene.

### 4.2 Cloning eIF3 subunits into pBIG1

Individual eIF3 subunits were cloned into pBIG1 A–D vectors (Figure 1a). Subunits k, l, e, and f were subcloned into pBIG1A. Subunits h, d, and m were cloned into pBIG1B. Subunits a and c were cloned together into pBIG1C, as they form the eukaryotic conserved eIF3 core. Subunits b, g, and i, for the conserved eIF3bgi module, were cloned into pBIG1D.

For the subcloning into pBIG1 vectors we followed the protocol described for biGBac cloning (Weissmann et al., 2016). A PCR using Phusion High‐Fidelity PCR Master Mix (Thermo Fisher Scientific, F351L) was performed for each individual subunit with the corresponding set of Cas forward/reverse primers based on the order of each subunit in the final pBIG1 vector (Figure 1a). The primers amplified the entire insect cell expression cassette (i.e., from Polyhedrin promoter to SV40 polyA sequence) and contained the linker sequences for subsequent Gibson assembly. PCRs were performed following manufacturer's protocol, in a final volume of 50 μL and a total amount of 50 ng DNA template with 2 min 30 s of extension time for all the subunits. For degradation of the parental vectors, the obtained PCR products were directly supplemented with 1 U of *Dpn*I enzyme (Promega, R6231), incubated for 1 h at 37°C, and purified with a Qiagen PCR purification kit (Qiagen, 28104). The purified PCR products were analyzed using a 1% agarose gel to ensure correct amplification (Figure S2).

About 10 μg of pBIG1 plasmids (A, B, C, and D) were linearized with 20 U of *Swa*I restriction enzyme (NEB, R0604L) for 2 h at 25°C. Correct linearization was checked on an analytical agarose gel and the linearized plasmid was purified with DNA purification Qiagen columns.

The pBIG1 vectors and the inserts were assembled with Gibson assembly master mix (NEB, E2611L). Corresponding PCR products for each subunit were mixed with pBIG1 vectors according to the plasmid design (Figure 1a), in a 1:1 insert:vector molar ratio. All inserts and the linearized vector were diluted to a final concentration of 25 nM and 1 μL of each insert and plasmid were mixed with 5 μL of Gibson master mix in a final volume of 10 μL (H_2_O was added when needed to adjust the final volume). Gibson reactions were incubated for 1 h at 50°C and 1 μL of each reaction was directly transformed into home‐made chemically competent *E. coli* TOP10 cells, following standard transformation protocols and using gentamycin as a selection antibiotic.

Plasmids isolated from the colonies after transformation were digested with *Bam*HI and *Xba*I restriction enzymes (NEB, R3136M, and R0145M, respectively) for 1 h at 37°C. The digestion results in excision of each original gene expression cassette. Digestion products were analyzed on a 1% agarose gel to identify positive clones (Figure S2b). Clones containing all the subunits were further sequenced, and correct clones were used to generate the pBIG2 vectors. At this point each pBIG1‐eIF3 construct can be used to generate bacmids to independently express a set of subunits in insect cells. All pBIG1 sequences and plasmids are available at Addgene.

### 4.3 Cloning pBIG1 multi‐gene cassettes into pBIG2

The pBIG1 vectors were combined to generate two final eIF3 pBIG2 vectors (AB and CD, Figure 1b), or one pBIG2ABC_eIF3‐∆bgi plasmid (Figure 5a) following the same strategy described in the original biGBac system paper (Weissmann et al., 2016). Fifty microgram of pBIG2 plasmids (AB, CD, or ABC) were linearized with 20 U of *Pme*I restriction enzyme (NEB, R0560L) for 2 h at 37°C and run on a 1% agarose gel. The linearized product band was cut from the gel and purified using QIAquick Gel Extraction Kit (Qiagen, 28704).

To optimize the cloning results, we coupled the excision of the multi‐gene cassettes containing eIF3 subunits with the Gibson reaction. The final ratio used for the Gibson reaction was 1:5 pBIG2:pBIG1x molar ratio of the plasmids. The pBIG1 plasmids were premixed in equimolar amounts (pBIG1A and B, and pBIG1C and D for the first cloning strategy, pBIG1A, B and C for the second) in a final volume reaction of 9 μL, then 20 U of *Pme*I was added for excision of the multi‐gene cassettes. After 2 h of incubation at 37°C, 1 μL of corresponding *Pme*I‐linearized pBIG2 plasmid (concentration adjusted to meet 1:5 molar ratio with the inserts) and 10 μL of NEBuilder HiFi DNA assembly master mix (NEB, E2621L) were added directly to the digestion reaction. The reaction was incubated for 1 h at 50°C. After the incubation, 5 μL of each reaction was transformed into High‐efficiency NEB 10‐beta Competent *E. coli* cells (NEB, C3019I) following manufacturer's instructions and allowing 16 h of recovery at 37°C with agitation. Cells were plated on chloramphenicol selection plates and grown until colonies appeared.

Small colonies, indicative of slow growth due to amplification of a large plasmid, were picked and plasmids amplified. The clones were analyzed as described before, on a 1% agarose gel after *Xba*I and *Bam*HI restriction digestion. The clones with all eIF3 subunits (Figure S2c) were selected and their sequences further confirmed by whole‐plasmid sequencing.

### 4.4 Baculovirus generation

In‐house prepared DH10Bac cells incorporating a YFP expression cassette, were used to generate bacmids for baculovirus generation. About 1–40 ng of each pBIG2 plasmid, or pBIG1D_eIF3bgi, respectively, was transformed in 50 μL of DH10Bac following standard procedures and allowing a recovery of 4 h at 37°C shaking. Cells were plated on corresponding LB/Kan/Gen/Tet/IPTG/BluO‐gal selection plates and incubated at 37°C for 2 days (or until clones appeared on the plate). Positive (white) colonies were grown, and bacmids were purified by alkaline lysis followed by isopropanol precipitation. Due to the large size of the bacmids, normal DNA purification columns are inefficient. The bacmids were stored at 4°C (avoid freezing) for no longer than 1 month.

Each baculovirus was prepared separately. Sf9 insect cell line (ATCC, CRL‐1711) was used to generate the baculoviruses. Two milliliter of 5 × 10^5^ Sf9 cells in mid‐log phase, growing in Sf‐900 II SFM insect cell media (Thermo Fisher, 10902104) were plated per well of a 6‐well TC well plate (Corning, 3516) and incubated for at least 30 min at 27°C without agitation to allow the cells to settle at the bottom of the plate. Bacmids (~1–5 μg) were transfected using FuGENE 6 or FuGene HD transfection reagent (Promega, E2691 or E2311) following manufacturer's instructions.

Cells were incubated at 27°C without shaking between 5 and 7 days, and fluorescence was checked regularly. When 100% of cells were YFP positive and cells started to detach, indicating a decrease in cell viability, cells and supernatant were collected by pipetting up and down. The supernatant was cleared by centrifugation at 300*g*, 4°C for 5 min and the supernatant containing the first generation of the baculovirus (P0) was used to generate the next baculovirus generation (P1).

0.5 mL of P0 virus was used to infect 50 mL of Sf9 cells at 1–2 × 10^6^ cells/mL (1:100 volume ratio virus:cells). Cells were incubated at 27°C and 140 rpm shaking for 2–3 days. Fluorescence and cell viability were checked every day by microscopy (example in Figure 2a). Virus was harvested when 100% of cells fluoresced, and viability was around 50%. To harvest the virus, infected cells were spun at 800*g* for 10 min at 4°C. The supernatant containing the second generation of the baculovirus (P1) was stored at 4°C protected from light until protein expression. Baculovirus were stored for no longer than 2 weeks. Viral titre was not measured, and further infections were optimized based on volume:volume of virus/insect cells ratio.

It is advisable to check for correct expression of some eIF3 subunits before large‐scale expression by saving the pellet after harvesting of the P1 viruses and performing an SDS‐PAGE or a Western Blot using antibodies against any of the eIF3 human subunits or any of the TAGs included in the constructs. For instance, expression and solubility of the eIF3‐∆bgi and eIF3bgi subcomplexes (Figure Sa) were checked by resuspending cells from P1 virus generation in a lysis buffer containing 20 mM HEPES/KOH (pH 7.5), 150 mM KCl, 10% glycerol, 1 mM DTT, and 1% Triton X‐100, followed by brief vortexing. The lysate was then cleared by centrifugation and the pellet (containing insoluble material) and supernatant were analyzed by SDS‐PAGE and Coomassie staining.

### 4.5 Protein expression optimization

Protein was expressed in the High Five (BTI‐Tn‐5B1‐4) insect cell line. When cells were co‐infected with the two viruses obtained from pBIG2AB/CD, protein expression was optimized by adjusting the virus ratio and cell harvesting time post‐infection (Figure S3). To test different conditions, 200 mL of Hi5 cells were co‐infected at a ratio of 1:50 or 1:100 (virus volume:Hi5 cells volume) with the mixture 1:1 of the two eIF3 bacmids and cells were incubated at 27°C at 140 rpm. Every 24 h an aliquot of 50 mL of cells was harvested and centrifuged for 5 min at 300 rpm, 4°C. The cell pellet was flash frozen in LN2 until further use.

To test the expression and assembly of the complex, cell pellets were lysed using hypotonic lysis buffer (20 mM Hepes‐KOH pH 7.5, 10 mM KCl, 1.5 mM MgCl_2_, 1 mM DTT, 1 mM PMSF, 1 tablet of EDTA‐free protease inhibitor cocktail (MERK, 11836170001) per mL of buffer and 50 U/mL DNAse1) in a 1:3 ratio (g cells/lysis buffer volume) and passed through a 24G needle 4 times. The lysate was cleared by centrifugation at 20,000*g* at 4°C for 10 min. The supernatant was saved and mixed with 50 μL of MagStrep Strep‐Tactin XT beads (IBA‐2‐5090‐050; 50 μL of bead suspension pre‐equilibrated in lysis buffer for 50 mL initial cell culture volume). Lysates and beads mixtures were incubated for 2 h at 4°C with rotation. Samples were placed into a magnetic rack and 20 μL of each supernatant was saved for further analysis (Figure S6). Beads were washed 3 times with buffer A (20 mM Hepes‐KOH pH 7.5, 100 mM KCl, 10% glycerol (v/v), 1 mM DTT), saving 20 μL of the last wash for further analysis, and eIF3 was then eluted with SDS‐PAGE sample buffer (50 mM Tris–HCl, 100 mM DTT, 2% SDS (w/v), 1.5 mM bromophenol blue and 1M glycerol). For complete elution, the tube was heated at 95°C for 2 min and the sample was separated from the beads by placing the tube in a magnetic rack. The flowthrough (F), wash (W) and elution (E) of all the samples were analyzed by SDS‐PAGE followed by Coomassie staining (Figure S3). In all cases we were able to recover all the subunits of eIF3 indicating that the complex was fully assembled. As this affinity purification is only selecting from the Strep‐tag, elution fractions were enriched in the subunit containing the strep TAG (eIF3c) and the closer binding partners, as expected. The best expression levels were observed at a virus:Hi5 cells volumetric ratio of 1:50 (co‐infection with the two pBIG2AB/CD viruses, 1:50 ratio each) or 1:75 (infection with pBIG1D_eIF3bgi/pBIG2ABC_eIF3‐∆bgi) and 48 h expression. Cells harvested at earlier timepoints presented a lower expression level, while later timepoints started to show some degradation of the factors. We thus used these optimized conditions for protein expression.

For large scale expression, both pBIG2AB/CD viruses were mixed in a 1:1 volume ratio and the viral mixture was used to infect High Five insect cells at a density of 1.5 × 10^6^ cells/mL and a ratio of 1:50 (virus/cells volume). Cells were incubated for 48 h at 27°C, 140 rpm shaking. Cell viability and fluorescence were checked regularly. Cell viability should be over 95% at the point of harvesting to avoid protein degradation. After 48 h, cells were spun at 300*g* for 10 min at 4°C. The supernatant was discarded, and the cell pellet was washed with ice‐cold PBS. Cells were pelleted again at 300*g* for 10 min at 4°C; the supernatant was discarded, and the pellet was flash‐frozen in LN2 and stored at −80°C.

Expression of isolated eIF3‐∆bgi and eIF3bgi subcomplexes was performed as described for FL eIF3, except that High Five cells were infected separately with corresponding eIF3‐∆bgi or eIF3bgi P1 viruses at a 1:75 ratio (virus:cells, v/v). Cells were harvested 51–55 h post‐infection as described for FL eIF3 and kept as separate pellets at −70°C.

### 4.6 Protein purification

Frozen cell pellets were incubated at 25°C water bath until completely thawed (between 5 and 10 min). The pellets were resuspended in hypotonic lysis buffer in a 1:3 weight:volume ratio (g cells/mL lysis buffer). Hypotonic lysis buffer consists of 20 mM Hepes‐KOH pH 7.5, 10 mM KCl, 1.5 mM MgCl_2_, 1 mM DTT, 1 mM PMSF, 1 tablet of EDTA‐free protease inhibitor cocktail (MERK, 11836170001)/50 mL of buffer and 50 U/mL DNAse I. 2 mL of Biolock (iba, 2‐0205‐050) per 1 L final insect cell culture volume was added to reduce unspecific binding of contaminants during strep‐tag affinity purification.

Pellets were resuspended in the lysis buffer by stirring at 4°C for 5 min. Cells were further lysed in a pre‐cooled Dounce homogenizer by 25 strokes. We found that this lysis method was required to limit proteolytic degradation of the large eIF3 subunits due to release of lytic enzymes by, for example, sonication. Once lysed, glycerol was added to a final concentration of 10% and KCl concentration was adjusted to final 100 mM to help with the stability of the complex. From this step everything was performed at 4°C (ice or cold room).

Lysate was clarified by centrifugation at 35,000*g* for 30 min at 4°C. Supernatant was saved and filtered through 0.45 μm filter before proceeding with the purification.

We used Strep‐Tag affinity purification in a first step as it is the most efficient in terms of cost/protein recovery, and the columns are easy to regenerate and can be reused for many purifications.

A 5 mL StrepTrap XT column (Cytiva, 29401320) was pre‐equilibrated with buffer A (20 mM Hepes‐KOH pH 7.5, 100 mM KCl, 10% glycerol (v/v), 1 mM DTT) operated at an ÄKTA pure. The filtered lysate was loaded onto the column at 3 mL/min, washed with buffer A until both 260 and 280 nm absorbance reached basal levels. Then, the column was washed with at least five column volumes (CV) of wash buffer (20 mM Hepes‐KOH pH 7.5, 300 mM KCl, 10% glycerol (v/v), 1 mM DTT) and re‐equilibrated back with at least five CV of buffer A. Protein was eluted with 50 mM biotin in buffer A at 3 mL/min.

For the test purifications, 2 mL fractions were collected and further analyzed on a 4–12% SDS‐PAGE followed by Coomassie staining (Figure S5a).

The eIF3 complex contains several RNA binding domains (Hayek et al., 2021; Sun et al., 2013), and the complex eluted from the Strep Trap XT column is highly contaminated with nucleic acids, as monitored by 260 nm/280 nm absorbance ratio (Figure S5b). A Heparin column was used to remove RNA contaminations and further purify the complex. The eluate from the affinity purification was loaded onto a 5 mL HiTrap Heparin HP column (Cytiva 17040701) equilibrated in buffer A, at 3 mL/min. The absorbance at 260 and 280 nm was recorded. The Heparin column was washed with buffer A until the absorbance measurement returned to basal levels, and the protein was eluted in a 100–500 mM KCl gradient (in buffer A) over 75 mL; 1.5 mL fractions were collected. RNA contamination was removed after the Heparin column step (Figure S5b). For routine purifications, protein can be directly eluted from the StrepTrap onto the Heparin column (in‐tandem purification).

Fractions from the Heparin column were analyzed by SDS‐PAGE followed by Coomassie staining (Figure S5b). In every case, several subcomplexes were observed in addition to the complete eIF3 complex. This could be due to degradation, differential expression or aberrant assembly. To further separate the subcomplexes from the complete eIF3 complex, the fractions containing all the eIF3 subunits were combined and concentrated using a 100 kDa MWCO concentrator (Amicon) to a suitable volume in preparation for size exclusion chromatography (SEC). The concentrated protein was injected onto erose 6 Increase 10/300 GL column (Cytiva 29091596), pre‐equilibrated in protein storage buffer (20 mM Hepes‐KOH pH 7.5, 150 mM KCl, 10% glycerol, 1 mM DTT) and 1 mL fractions were collected. The fractions were analyzed by SDS‐PAGE followed by Coomassie staining (Figure S5b). Fractions containing the complete eIF3 complex were pooled and concentrated using a 100 kDa MWCO concentrator until the desired volume/concentration was reached. Final concentrations of eIF3 are indicated for each method. The eIF3 complex was aliquoted, flash frozen in liquid nitrogen and stored at −80°C for further analysis.

For purification of the eIF3‐∆bgi complex, cells from 1.5 L culture (~22 g) were thawed in a room temperature water bath, then resuspended in 100 mL of hypotonic lysis buffer (20 mM HEPES/KOH, 1.3 mM Mg(OAc)_2_, 10 mM KCl, 1 mM DTT (pH 7.5)) supplemented with one cOmplete Protease Inhibitor Cocktail tablet (EDTA‐free; Roche).

For purification of FL eIF3 from individually expressed eIF3‐∆bgi and eIF3bgi, cells having expressed the individual subcomplexes were mixed at eIF3‐∆bgi:eIF3bgi pellet mass ratio of 1.5:1 (30 g:20 g, corresponding to cells from 1 L vs. 2 L cultures, respectively), resuspended in a total of 120 mL hypotonic lysis buffer supplemented with three cOmplete Protease Inhibitor Cocktail tablets (EDTA‐free; Roche) and 1 mM phenyl methyl sulfonyl fluoride (PMSF; APExBIO, Cat. #A2587).

In both cases, the complexes were purified as described above. After final SEC, proteins were concentrated to 36 μM/22 mg/mL (eIF3‐∆bgi, 2.2 mg final yield), or 25.5 μM/20 mg/mL (FL eIF3, 10 mg final yield), respectively, aliquoted, then shock‐frozen using liquid nitrogen and stored at −70°C.

For purification of isolated eIF3bgi, cells from a 1 L expression (~20 g cell pellet) were thawed in a RT water bath, then resuspended in 60 mL lysis buffer (40 mM Tris/HCl, 500 mM NaCl, 5% Glycerol (pH 7.4)) and one cOmplete Protease Inhibitor Cocktail tablet (EDTA‐free; Roche) was added. The cells were lysed using sonication and the lysate was then cleared by centrifugation (40 min at 18,000 rpm, 4°C in a JA 25.50 rotor) and filtering through a 0.22 μm syringe filter. The raw extract was then loaded to a 100 mL gravity flow column containing 10 mL of Ni‐NTA resin (Qiagen, Cat. #30210) and the column was rolled for 1 h at 4°C to allow binding of the eIF3bgi complex via the C‐terminal His10 tag on eIF3i. The column was subsequently washed with high salt buffer (40 mM Tris/HCl, 1M NaCl (pH 7.4)) and the complex then eluted sequentially using 20 mL of buffer A (40 mM Tris/HCl, 50 mM KCl, 5% glycerol (pH 7.4)) containing 50, 150, or 300 mM imidazole, respectively. The three elution fractions were combined, then loaded to 5 mL HiTrap Heparin column (Cytiva, Cat. #17040601) equilibrated in buffer A at 2 mL/min. The column was washed with 20 mL buffer A (3 mL/min) and the complex subsequently eluted using a constant gradient from buffer A to buffer B (40 mM Tris/HCl, 1M KCl, 5% glycerol (pH 7.4)) over 30 mL. Fractions containing the target proteins were combined, concentrated via ultrafiltration and then applied to a Superose6 Increase 10/300 GL SEC column (Cytiva, Cat. #29091596), equilibrated in buffer C (40 mM Tris/HCl, 30 mM KCl, 5% glycerol, 2 mM DTT (pH 7.4)). The complex was eluted at 0.6 mL/min. Target fractions (0.5 mL) were combined, concentrated to 165 μM (~27 mg/mL) via ultrafiltration, aliquoted and then shock‐frozen using liquid nitrogen and stored at −70°C. The final yield was 2.7 mg.

### 4.7 Integrity and activity test of recombinant eIF3

#### 4.7.1 Mass spectrometry

Purified eIF3 (1 μL of ~25 μM) was diluted with 10 μL 100 mM NH_4_HCO_3_, 5 μL of 1% Rapigest in 50 mM NH_4_HCO_3_ was added and the sample heated for 10 min at 80°C. Then 5 μL DDT (4 mM final) and iodoacetamide (14 mM final) were added, and the sample incubated for 30 min at RT in the dark. The sample was further diluted with 40 μL 100 mM NH_4_HCO_3_, then 2 μL of 0.5 μg/μL trypsin was added and the proteins digested at 37°C. The sample was then acidified by addition of trifluoroacetic acid (0.5% final); 20 μL were then desalted using a C18 peptide desalting tip (elution buffer: 25% H_2_O + 70% acetonitrile +0.1% formic acid). The eluted peptides were then dried using a SpeedVac and dissolved in 0.1% formic acid, 3% acetonitrile.

The extracted tryptic peptides (2 μL) were analyzed by nano‐scale capillary LC–MS/MS using an Ultimate U3000 HPLC (Thermo Fisher Scientific). The sample was loaded onto the trapping column (Thermo Scientific, PepMap100, C18, 300 μm × 5 mm) and then resolved on the analytical column (Aurora 15 cm column) at a flow rate of 300 nL/min using a gradient of 100% A (0.1% formic acid) to 40% B (80% acetonitrile 0.1% formic acid) over 40 min.

Peptides were analyzed with a hybrid linear quadrupole ion trap mass spectrometer (Orbitrap QExactive plus, Thermo Fisher Scientific). Data dependent analysis was carried out using a resolution of 70,000 for the full MS spectrum, followed by 10 MS/MS spectra in the linear ion trap. MS spectra were collected over a m/z range of 200–1800. MS/MS scans were collected using threshold energy of 35 for collision induced dissociation. MS spectra were identified using the SequestHF algorithm for a database search against the UniProt reference proteome for *Homo sapiens* in Proteome Discoverer v3.0 software (Thermo Fisher Scientific).

#### 4.7.2 Western blot

Samples were loaded onto a NuPAGE 4–12% Bis‐Tris Gel (Invitrogen NP0323BOX). Electrophoresis was performed for 1 h at 150 V. Proteins were transferred to a PVDF membrane using a Bio‐Rad trans‐turbo blotting system.

After transfer, the membranes were washed 3 times with PBS 0.1% Tween‐20 and blocked for 1 h at RT with PBS 5% milk. Antibodies used in this study are α‐FLAG (F1804, 1:1000), α‐strep (PA5‐119772, 1:10,000), α‐HA (REF, 1:1000), α‐eIF3a (Ab128996, 1:5000), α‐eIF3b (Ab133601, 1:20,000), α‐eIF3c (REF, 1:1000), and α‐eIF3g (Ab192601, 1:2000). Antibodies were incubated in PBS 5% milk overnight at 4°C. The membranes were washed 3 times (5 min at RT with shaking) with PBS 0.1% Tween‐20 and incubated 1:20000 (in PBS 5% milk) with the respective fluorescent‐dye conjugated secondary antibodies (α‐Rabbit‐800 nm, A32735; α‐Rabbit‐680 nm, A32734; α‐mouse 800 nm, A32730). After incubation, membranes were washed 2 times with PBS 0.1% Tween‐20 and one final wash with PBS1X. Membranes were directly imaged in a ChemiDoc MP imaging system (BioRad).

#### 4.7.3 RelE assay

RelE assay was performed essentially following the original protocol (Querido et al., 2020). Human initiation factors, ribosomes, capped mRNA and tRNA_i_^Met^ were prepared as described previously (Feoktistova et al., 2013; Sokabe & Fraser, 2014; Sokabe & Fraser, 2017). 43S PIC (300 nM eIF1/1A/5/2, 350 nM Met‐tRNA_i_, 200 μM GMPPNP, 200 nM 40S subunit, and 250 nM different eIF3 complexes) was prepared in ribosome buffer (20 mM HEPES pH 7.5, 70 mM KCl, 2.5 mM MgCl_2_, 0.1 mM spermidine, 1 mM DTT, and 10% glycerol) and preincubated for 5 min at 37°C. The eIF4F complex was assembled in ribosome buffer and consisted of 500 nM eIF4G (residues 557–1599)/eIF4A/eIF4E mixed with 50 nM of 5′ capped and 3′ fluorescein‐labeled mRNA and 0.5 mM ATP‐Mg. The 4F complex was incubated for 5 min at 37°C. Both the 43S PIC and eIF4F complexes were mixed (final volume 10 μL) and incubated for a further 5 min at 37°C. After incubation, 8 μM RelE enzyme was added, and the mixture was incubated for 10 min at 37°C. The reaction was quenched by adding 10 μL of 8M urea. The RNA was resolved in denaturing conditions by 10% polyacrylamide gel with 8M urea and imaged by a Typhoon analyser (Cytiva).

#### 4.7.4 Toe‐printing assay

Toe‐printing (TP) assays were performed as described previously (Pisarev et al., 2007). Human translation initiation factors, 40S subunits and mRNA/tRNA (prepared as described previously; Feoktistova et al., 2013; Sokabe & Fraser, 2014; Sokabe & Fraser, 2017) were prediluted in TP buffer (25 mM HEPES/KOH, 100 mM KOAc, 2.5 mM MgCl_2_, 0.25 mM spermidine, 1 mM DTT (pH 7.5)). The test mRNA contained a 5′ cap and 55 nt synthetic 5′ UTR, followed by an AUG codon in a good Kozak context. Toe‐printing samples (15 μL final volume) contained 50 nM mRNA, 0.5 U/μL RNaseIN, and 0.5 mM ATP. For all samples except the negative controls, the mRNA was activated by the addition of eIF4F/4B (250 nM eIF4G (165‐1599), 500 nM eIF4A, 250 nM eIF4E, 250 nM eIF4B; all final concentrations). A 43S PIC was prepared by mixing 40S subunits (100 nM), eIF1, eIF1A (both 500 nM), eIF2 (250 nM), eIF5 (200 nM), Met‐tRNA_i_^Met^ (200 nM), 0.5 mM GTP, and as applicable, FL eIF3 (native or recombinant, 200 nM), eIF3‐∆bgi (200 nM), eIF3bgi (200 nM), or reconstituted eIF3 (obtained by mixing each 200 nM eIF3‐∆bgi and eIF3bgi, and incubation for 5 min at 30°C) and preincubation for 10 min at 30°C. The 43S PIC was then added to (activated) mRNA and initiation allowed to occur for 15 min at 37°C. After incubation, 5 μL of reverse transcription (RT) master mix containing 5 U of AMV reverse transcriptase, 1X AMV reverse transcriptase buffer (both Promega, Cat. #M5101), 0.5 mM dNTPs mix, 5 mM MgCl_2_, and 100 nM of a 5′ Yakima Yellow labeled primer (annealing 111 nt downstream of AUG‐55 codon) was added (20 μL final volume). The RT reaction was carried out for 45 min at 37°C under dark conditions. The sample volume was then adjusted to 100 μL with TES buffer (10 mM Tris/HCl (pH 8.0), 10 mM EDTA, 0.5% SDS) and cDNAs were subsequently Phenol:Chlorophorm‐extracted, EtOH precipitated and finally redissolved in 3.5 μL of formamide‐EDTA loading dye. The samples were then loaded to 0.4 mm, 7M urea, 6% acrylamide sequencing gels next to sequencing ladders prepared using the RT primer and the USB Thermo Sequenase Dye Primer Manual Cycle Sequencing Kit (Affymetrix, Inc., Cat. #79260). Electrophoresis was carried out at RT for 2 h at 30 W constant power, and under dark conditions. Bands were then visualized using a Typhoon imager (Cytiva).

#### 4.7.5 Mass photometry

All mass photometry experiments were conducted using a Refeyn OneMP mass photometer (Refeyn Ltd.). Proteins (native or recombinant eIF3, eIF3‐∆bgi and eIF3bgi) were prediluted to 500 nM using MP buffer (25 mM HEPES/KOH, 150 mM KCl, 10% glycerol, 0.5 mM Tris(2‐carboxyethyl) phosphine hydrochloride (TCEP) (pH 7.5)). For the interaction experiment of eIF3‐∆bgi with eIF3bgi, the two pre‐diluted subcomplexes were mixed at equimolar ratios, then incubated for 10 min at 30°C. Just prior to measurement, proteins were further diluted to 50 nM (native/recombinant FL eIF3, eIF3‐∆bgi + eIF3bgi) or 100 nM (eIF3bgi) and centrifuged for 4 min at 15,000 rpm, 4°C. Six‐well silicon gaskets were positioned onto MassGlass UC coverslips (Refeyn), which were then placed on top of a drop of IMMOIL‐F30CC immersion oil (Olympus) on the objective lens. About 15 μL of sample was then applied to a well, the surface was brought into focus, and a 1 min movie was acquired in “Standard measurement” mode optimized for large proteins. Data analysis was done using the Refeyn DiscoverMP software v2024 R2 (Refeyn Ltd.). Ratiometric contrast was converted to molecular weight using a calibration curve acquired using MassFerence P1 Calibrant protein solution (Refeyn Inc., Cat. #MP‐CON‐41033) at 1:100 dilution.

#### 4.7.6 Analytical size exclusion chromatography

Analytical size exclusion chromatography (SEC) experiments were conducted using a Superose6 3.2/300 column operated at an ÄKTA pure micro (both Cytiva) at 4°C. Samples (FL eIF3, eIF3‐∆bgi, eIF3bgi, eIF3‐∆bgi + eIF3bgi) contained 10 μM protein buffered by SEC buffer (25 mM HEPES/KOH, 150 mM KCl, 10% glycerol, 0.5 mM TCEP (pH 7.5)) in a total volume of 25 μL. For reconstitution of FL eIF3 from eIF3‐∆bgi and eIF3bgi, the subcomplexes were incubated for 10 min at 30°C prior to the run. The samples were loaded to the column equilibrated in SEC buffer using a 50 μL loop and then eluted at 45 μL/min; 60 μL fractions were collected and analyzed by SDS‐PAGE/Coomassie staining. A mix of globular standard proteins (BioRad Gel Filtration standard (Cat. #1511901) + VitroEase Apoferritin (Thermo Fisher, Cat. #A51362)) was run to correlate elution volume with apparent molecular weight.

## AUTHOR CONTRIBUTIONS

**Irene Diaz‐Lopez:** Conceptualization; investigation; writing – original draft; methodology; validation; visualization; formal analysis; data curation. **Yuliya Gordiyenko:** Investigation; methodology; writing – review and editing. **Philipp K. Zuber:** Formal analysis; investigation; methodology; writing – review and editing. **Xueyan Li:** Investigation. **V. Ramakrishnan:** Conceptualization; funding acquisition; writing – review and editing; project administration; supervision; resources.

## CONFLICT OF INTEREST STATEMENT

The authors declare no conflicts of interest.

## Supporting information


**Figure S1.** Information about eIF3 subunits. (a) Corresponding Uniprot ID used in this study to extract the aminoacidic sequence for each eIF3 subunit. The different tags when corresponding are indicated, describing the position on the subunits and if present, protease to remove the tags.

**Figure S2.** Check of the correct amplification and cloning of eIF3 subunits. (a) PCR extraction and amplification for each eIF3 coding sequence subunit. (b) Enzymatic digestion of the selected pBIG1‐eIF3 clones. Corresponding eIF3 subunits in each plasmid and the correct size are indicated. (c) Enzymatic digestion of the select pBIG2‐eIF3 clones. Corresponding subunits and sizes are indicated.

**Figure S3.** Optimization for virus ratio and expression time in Hi5 cells. Result of streptavidin beads purification of 20 mLs of Hi5 cells lysate infected with 1/50 (a) and 1/100 (b) virus mix/cells volume ratio. Coomassie stained gel of the flowthrough (F), first wash (W) and elution (E) of each timepoint post infection. eIF3 subunits are labeled based on molecular weight.

**Figure S4.** eIF3 purification. (a) Chromatogram of affinity purification, using a streptavidin affinity column, of eIF3 lysate (column volume 5 mL). The indicated fractions were analyzed on a Coomassie‐stained SDS‐PAGE gel. Fractions 3 to 14 were pulled and loaded on heparin column. (b) Chromatogram of affinity purification, using heparin HiTrap column (column volume 5 mL), of the eIF3 pulled fractions from streptavidin affinity chromatography. KCl concentration gradient used to elute the complex is shown in brown. Fractions 1 to 58 were pulled and loaded onto SEC column. (c) Chromatograms of SEC run using a Superose6 3.2/300 column of the pulled fractions from heparin column purification. Total column volume is 2.5 mL. Fractions 4 and 5 corresponding to the main peak of the chromatogram were pulled and consider as the full complex.

**Figure S5.** Additional analysis of eIF3‐∆bgi and eIF3bgi. (a) Coomassie‐stained SDS polyacrylamide gel showing the insoluble (pellet, P) and soluble (supernatant, SN) fractions of P1 virus generating cells. Unambiguously identified subunits are indicated on the right. (b) Chromatograms of analytical SEC runs using a Superose6 3.2/300 column of isolated eIF3‐∆bgi, eIF3bgi, a mix of the two subcomplexes at equimolar ratio (eIF3‐∆bgi + eIF3bgi), or complete eIF3 obtained by purification of mixed cell pellets. The asterisk marks the exclusion (void) volume. The molecular weights were determined from a run of globular standard proteins. All SEC samples contained 10 μM of the corresponding complexes in 25 μL. (c) Coomassie‐stained SDS polyacrylamide of the fractions from the four analytic SEC runs as highlighted in (b); gels are color‐coded as curves in (b). (d, e) Mass photometry histograms of eIF3bgi (theor. MW: 166 kDa), eIF3‐∆bgi (theor. MW: 609 kDa) native eIF3 (theor. MW: 764 kDa), and recombinant eIF3 (theor. MW: 775 kDa), acquired at the indicated concentrations. Masses of the subcomplexes were derived from a Gaussian fit. For the interaction experiment between eIF3‐∆bgi and eIF3bgi, the two subcomplexes were incubated at equimolar ratio at 500 nM for 10 min at 30°C, then diluted to 50 nM for measurement. (f). Chromatogram of analytical SEC runs using a Superose6 3.2/300 column of isolated eIF3‐∆bgi at KCl concentrations of 150 mM (same KCl concentration as in (b)), 250 or 400 mM, respectively. The asterisk marks the exclusion (void) volume. All SEC samples contained 1.5 μM protein in 25 μL.

**Figure S6.** Schematic representation of the eIF2 subunits organization in the plasmids. (a) Arrangement of individual eIF2 subunits in pACEBac plasmids and final combination on the pBIG1A plasmid. (b) Enzymatic digestion of the positive pBIG1A‐eIF2abg clone. PmeI digestion release the entire fragment containing the three subunits (5 Kb approx.) and XbaI + BamHI digestion releases individual subunits (sizes label on the panel).

**Table S1.** Mass spectrometry analysis of purified eIF3 complex. Result of the mass spectrometry analysis of the final eIF3 purifications, using as database the host proteome (*Trichoplusia ni*), adding human eIF3 subunits sequences. First tab corresponds to eIF3 complex purified with co‐infection with two bacmids strategy. Second tab corresponds to eIF3 complex purified mixing eIF3‐Δ‐bgi and eIF3 bgi (expressed separately).

## Data Availability

The data that support the findings of this study are openly available in Addgene at https://www.addgene.org/.
