# Supplementary material for: Purification and characterization of recombinant human translation initiation factor eIF3
Source: Protein Sci. 2025 Dec 23;35(1):e70388. doi: 10.1002/pro.70388 (PMC12723715; doi:10.1002/pro.70388)
Supplement: Supplementary file 1 — Figure S1. Information about eIF3 subunits. (a) Corresponding Uniprot ID used in this study to extract the aminoacidic sequence for each eIF3 subunit. The different tags when corresponding are indicated, describing the position on the subunits and if present, protease to remove the tags. [file PRO-35-e70388-s006.pdf]

A

SUBUNIT	Uniprot ID	TAG	TAG-position	TAG CLEAVAGE
eIF3a	Q15152	3xFLAG	C-terminal	TEV protease
eIF3b	P55884			
eIF3c	Q99613	3C_SCII	C-terminal	Precission protein
eIF3d	O15371	HA	C-terminal	
eIF3e	P60228			
eIF3f	O00303			
eIF3g	O75821			
eIF3h	O15372			
eIF3i	Q13347	10 HIS	C-terminal	
eIF3j	O75822			
eIF3k	Q9UBQ5			
eIF3l	Q9Y262			
eIF3m	Q7L2H7			

Figure S1. Information about eIF3 subunits. A. Corresponding Uniprot ID used in this study to extract the aminoacidic sequence for each eIF3 subunit. The different tags when corresponding are indicated, describing the position on the subunits and if present, protease to remove the tags.
