# Supplementary material for: Purification and characterization of recombinant human translation initiation factor eIF3
Source: Protein Sci. 2025 Dec 23;35(1):e70388. doi: 10.1002/pro.70388 (PMC12723715; doi:10.1002/pro.70388)
Supplement: Supplementary file 2 — Figure S2. Check of the correct amplification and cloning of eIF3 subunits. (a) PCR extraction and amplification for each eIF3 coding sequence subunit. (b) Enzymatic digestion of the selected pBIG1‐eIF3 clones. Corresponding eIF3 subunits in each plasmid and the correct size are indicated. (c) Enzymatic digestion of the select pBIG2‐eIF3 clones. Corresponding subunits and sizes are indicated. [file PRO-35-e70388-s007.pdf]

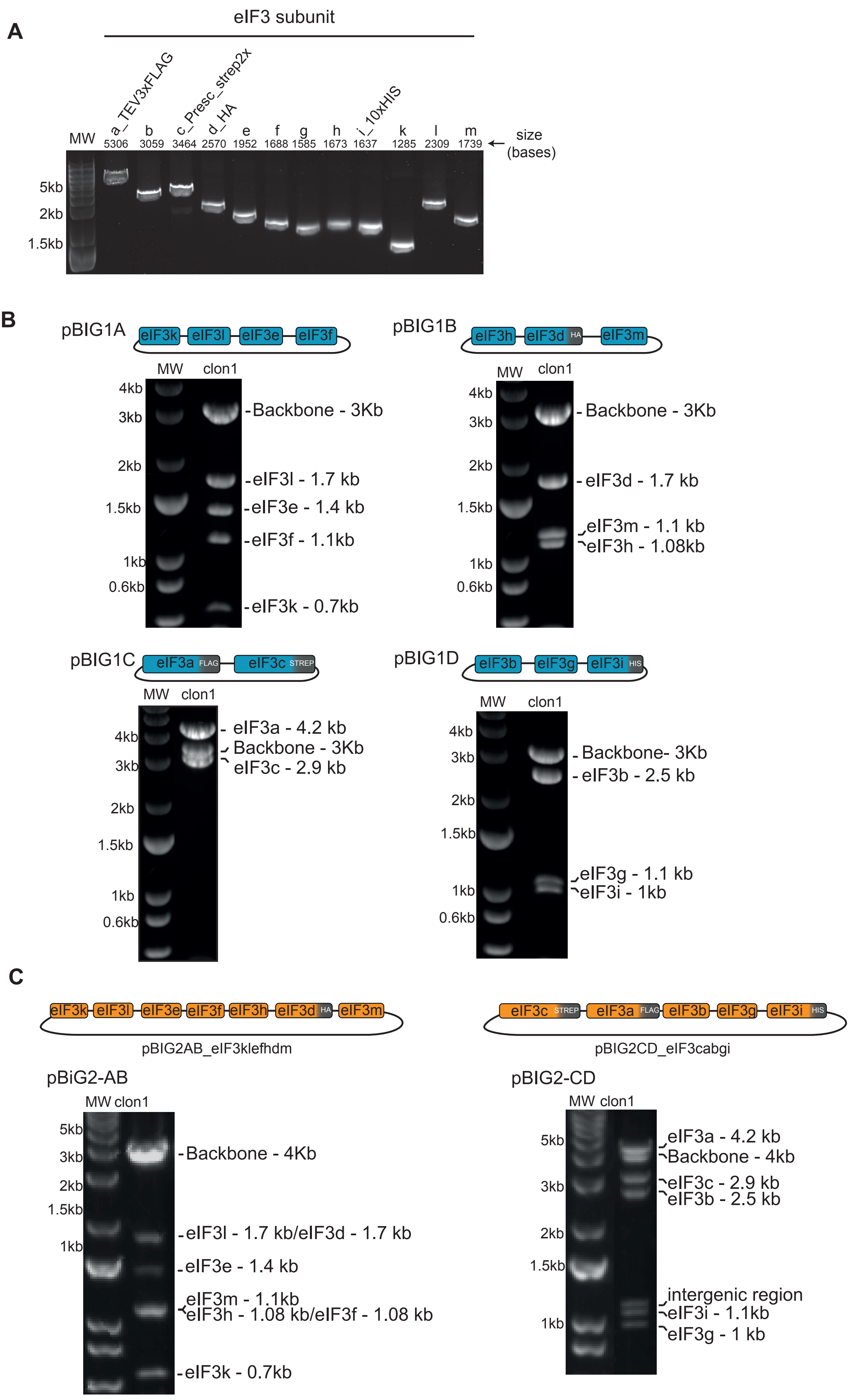
A
eIF3 subunit
MW
a_TEV3xFLAG
b
c_Presc_strep2x
d_HA
e
f
g
h
i_10xHIS
k
l
m
5306 3059 3464 2570 1952 1688 1585 1673 1637 1285 2309 1739
size (bases)
5kb
2kb
1.5kb
B
pBIG1A
eIF3k eIF3l eIF3e eIF3f
MW clon1
4kb
3kb
2kb
1.5kb
1kb
0.6kb
Backbone - 3Kb
eIF3l - 1.7 kb
eIF3e - 1.4 kb
eIF3f - 1.1kb
eIF3k - 0.7kb
pBIG1B
eIF3h eIF3d HA eIF3m
MW clon1
4kb
3kb
2kb
1.5kb
1kb
0.6kb
Backbone - 3Kb
eIF3d - 1.7 kb
eIF3m - 1.1 kb
eIF3h - 1.08kb
pBIG1C
eIF3a FLAG eIF3c STREP
MW clon1
4kb
3kb
2kb
1.5kb
1kb
0.6kb
eIF3a - 4.2 kb
Backbone - 3Kb
eIF3c - 2.9 kb
pBIG1D
eIF3b eIF3g eIF3i HIS
MW clon1
4kb
3kb
2kb
1.5kb
1kb
0.6kb
Backbone- 3Kb
eIF3b - 2.5 kb
eIF3g - 1.1 kb
eIF3i - 1kb
C
eIF3k eIF3l eIF3e eIF3f eIF3h eIF3d HA eIF3m
pBIG2AB_eIF3klefhdm
eIF3c STREP eIF3a FLAG eIF3b eIF3g eIF3i HIS
pBIG2CD_eIF3cabgi
pBiG2-AB
MW clon1
5kb
3kb
2kb
1.5kb
1kb
Backbone - 4Kb
eIF3l - 1.7 kb/eIF3d - 1.7 kb
eIF3e - 1.4 kb
eIF3m - 1.1kb
eIF3h - 1.08 kb/eIF3f - 1.08 kb
eIF3k - 0.7kb
pBIG2-CD
MW clon1
5kb
3kb
2kb
1.5kb
1kb
eIF3a - 4.2 kb
Backbone - 4kb
eIF3c - 2.9 kb
eIF3b - 2.5 kb
intergenic region
eIF3i - 1.1kb
eIF3g - 1 kb

Figure S2. Check of the correct amplification and cloning of eIF3 subunits. A. PCR extraction and amplification for each eIF3 coding sequence subunit. B. Enzymatic digestion of the selected pBIG1-eIF3 clones. Corresponding eIF3 subunits in each plasmid and the correct size are indicated. C. Enzymatic digestion of the selected pBIG2-eIF3 clones. Corresponding subunits and sizes are indicated.
