# Supplementary material for: Purification and characterization of recombinant human translation initiation factor eIF3
Source: Protein Sci. 2025 Dec 23;35(1):e70388. doi: 10.1002/pro.70388 (PMC12723715; doi:10.1002/pro.70388)
Supplement: Supplementary file 3 — Figure S3. Optimization for virus ratio and expression time in Hi5 cells. Result of streptavidin beads purification of 20 mLs of Hi5 cells lysate infected with 1/50 (a) and 1/100 (b) virus mix/cells volume ratio. Coomassie stained gel of the flowthrough (F), first wash (W) and elution (E) of each timepoint post infection. eIF3 subunits are labeled based on molecular weight. [file PRO-35-e70388-s003.pdf]

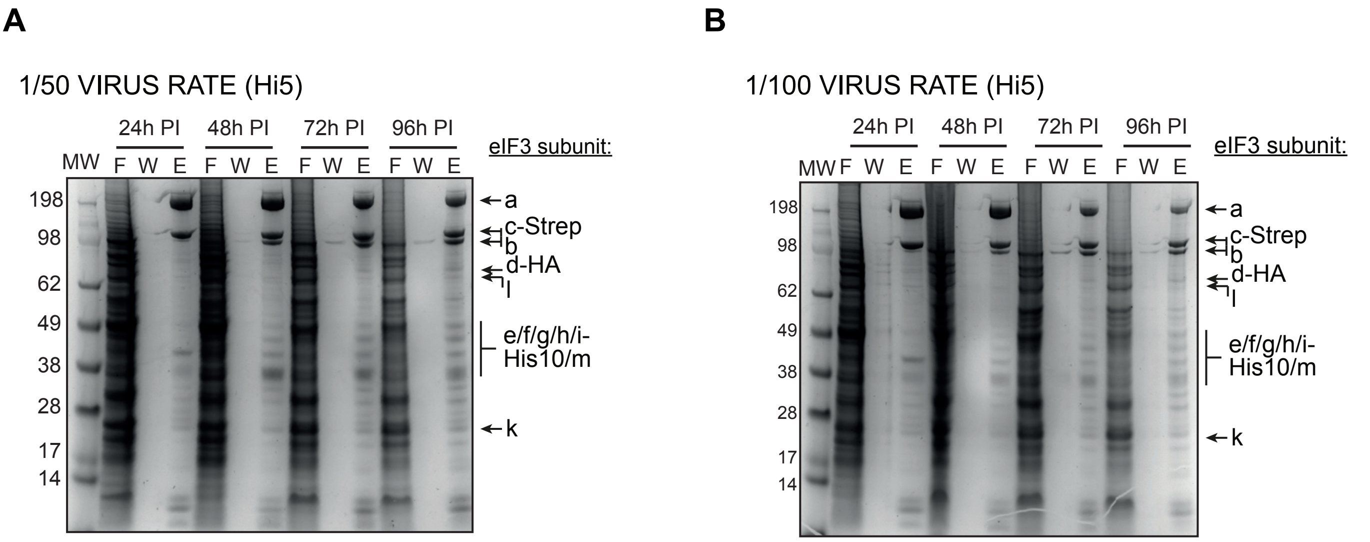

Figure S3. Optimization for virus ratio and expression time in Hi5 cells. Result of streptavidin beads purification of 20 mLs of Hi5 cells lysate infected with 1/50 (A) and 1/100 (B) virus mix/cells volume ratio. Coomassie stained gel of the flowthrough (F), first wash (W) and elution (E) of each timepoints post infection. eIF3 subunits are labelled based on molecular weight.
