# Supplementary material for: Purification and characterization of recombinant human translation initiation factor eIF3
Source: Protein Sci. 2025 Dec 23;35(1):e70388. doi: 10.1002/pro.70388 (PMC12723715; doi:10.1002/pro.70388)
Supplement: Supplementary file 4 — Figure S4. eIF3 purification. (a) Chromatogram of affinity purification, using a streptavidin affinity column, of eIF3 lysate (column volume 5 mL). The indicated fractions were analyzed on a Coomassie‐stained SDS‐PAGE gel. Fractions 3 to 14 were pulled and loaded on heparin column. (b) Chromatogram of affinity purification, using heparin HiTrap column (column volume 5 mL), of the eIF3 pulled fractions from streptavidin affinity chromatography. KCl concentration gradient used to elute the complex is shown in brown. Fractions 1 to 58 were pulled and loaded onto SEC column. (c) Chromatograms of SEC run using a Superose6 3.2/300 column of the pulled fractions from heparin column purification. Total column volume is 2.5 mL. Fractions 4 and 5 corresponding to the main peak of the chromatogram were pulled and consider as the full complex. [file PRO-35-e70388-s002.pdf]

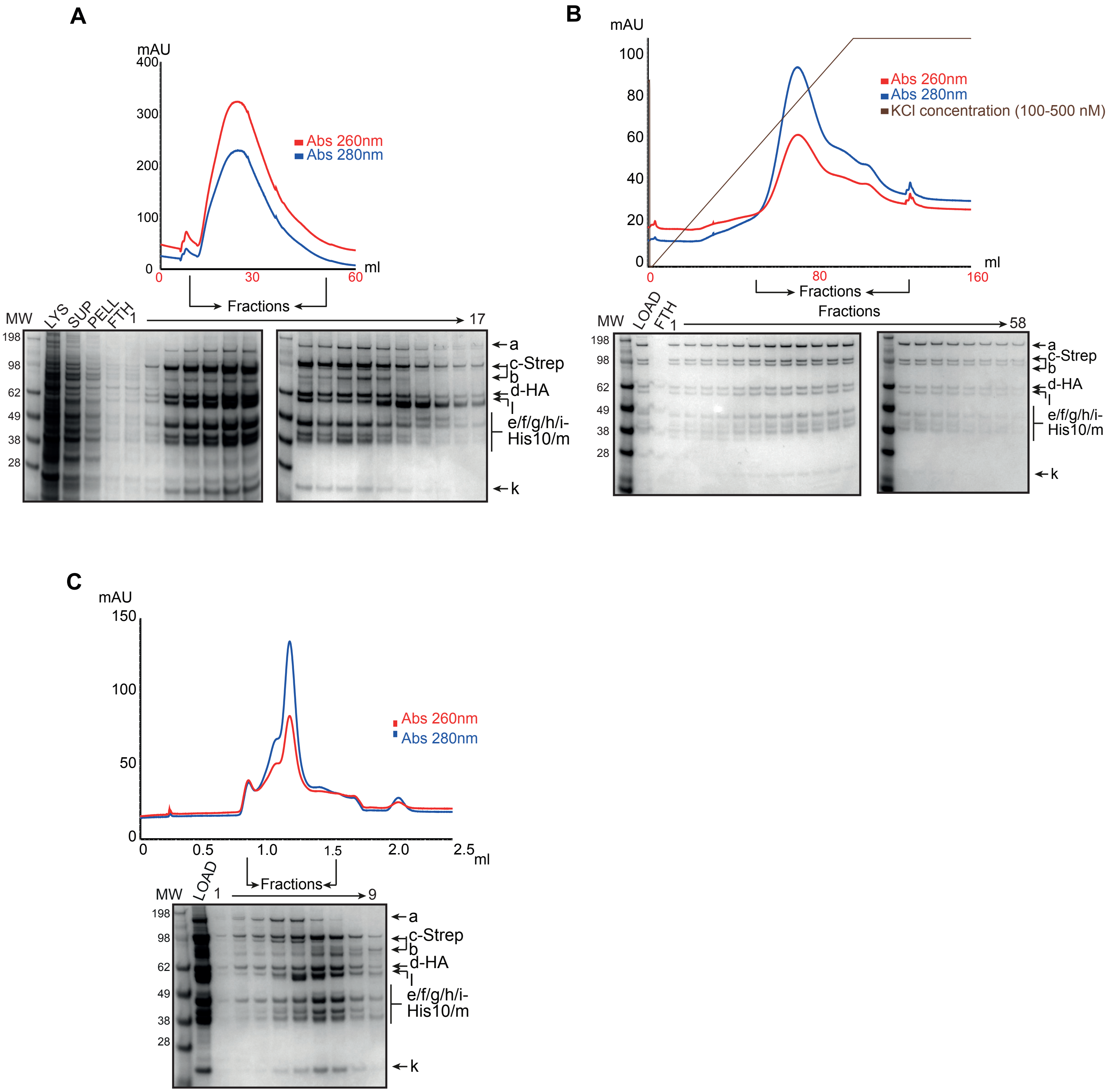

Figure S4. eIF3 purification. A. Chromatogram of affinity purification, using a streptavidin affinity column, of eIF3 lysate (column volume 5 mL). The indicated fractions were analysed on a Coomassie-stained SDS-PAGE gel. Fractions 3 to 14 were pulled and loaded on heparin column. B. Chromatogram of affinity purification, using heparin HiTrap column (column volume 5 mL), of the eIF3 pulled fractions from streptavidin affinity chromatography. KCl concentration gradient used to elute the complex is shown in brown. Fractions 1 to 58 were pulled and loaded onto SEC column. C. Chromatograms of SEC run using a Superose6 3.2/300 column of the pulled fractions from heparin column purification. Total column volume is 2.5 mL. Fractions 4 and 5 correcponding to the main peak of the chromatogram were pulled and consider as the full complex.
