# Supplementary material for: Purification and characterization of recombinant human translation initiation factor eIF3
Source: Protein Sci. 2025 Dec 23;35(1):e70388. doi: 10.1002/pro.70388 (PMC12723715; doi:10.1002/pro.70388)
Supplement: Supplementary file 5 — Figure S5. Additional analysis of eIF3‐∆bgi and eIF3bgi. (a) Coomassie‐stained SDS polyacrylamide gel showing the insoluble (pellet, P) and soluble (supernatant, SN) fractions of P1 virus generating cells. Unambiguously identified subunits are indicated on the right. (b) Chromatograms of analytical SEC runs using a Superose6 3.2/300 column of isolated eIF3‐∆bgi, eIF3bgi, a mix of the two subcomplexes at equimolar ratio (eIF3‐∆bgi + eIF3bgi), or complete eIF3 obtained by purification of mixed cell pellets. The asterisk marks the exclusion (void) volume. The molecular weights were determined from a run of globular standard proteins. All SEC samples contained 10 μM of the corresponding complexes in 25 μL. (c) Coomassie‐stained SDS polyacrylamide of the fractions from the four analytic SEC runs as highlighted in (b); gels are color‐coded as curves in (b). (d, e) Mass photometry histograms of eIF3bgi (theor. MW: 166 kDa), eIF3‐∆bgi (theor. MW: 609 kDa) native eIF3 (theor. MW: 764 kDa), and recombinant eIF3 (theor. MW: 775 kDa), acquired at the indicated concentrations. Masses of the subcomplexes were derived from a Gaussian fit. For the interaction experiment between eIF3‐∆bgi and eIF3bgi, the two subcomplexes were incubated at equimolar ratio at 500 nM for 10 min at 30°C, then diluted to 50 nM for measurement. (f). Chromatogram of analytical SEC runs using a Superose6 3.2/300 column of isolated eIF3‐∆bgi at KCl concentrations of 150 mM (same KCl concentration as in (b)), 250 or 400 mM, respectively. The asterisk marks the exclusion (void) volume. All SEC samples contained 1.5 μM protein in 25 μL. [file PRO-35-e70388-s005.pdf]

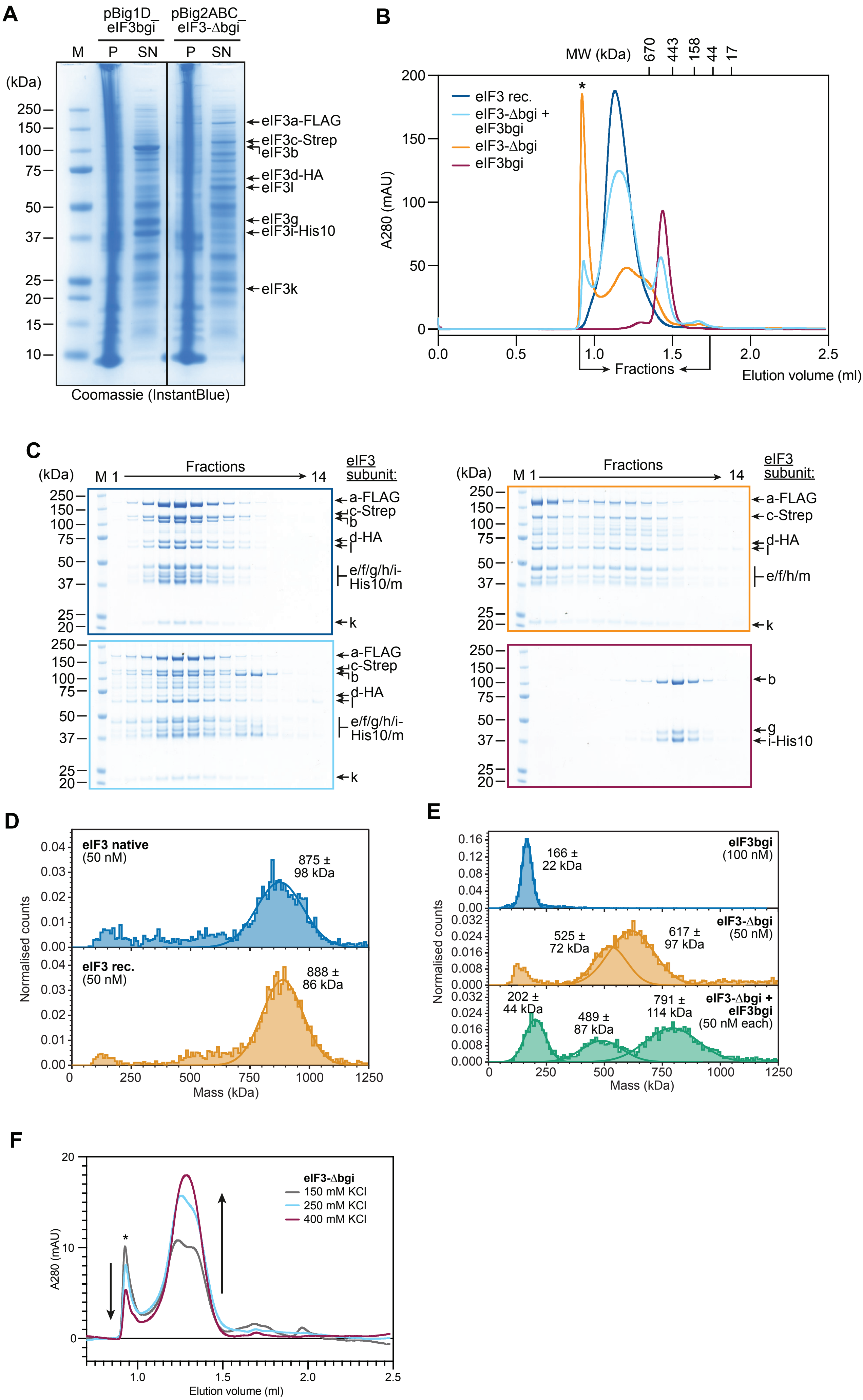
A
pBig1D_ eIF3bgi
pBig2ABC_ eIF3-Δbgi
M P SN P SN
(kDa)
250
150
100
75
50
37
25
20
15
10
eIF3a-FLAG
eIF3c-Strep
eIF3b
eIF3d-HA
eIF3l
eIF3g
eIF3i-His10
eIF3k
Coomassie (InstantBlue)
B
MW (kDa) 670 443 158 44 17
eIF3 rec.
eIF3-Δbgi + eIF3bgi
eIF3-Δbgi
eIF3bgi
A280 (mAU)
Fractions
Elution volume (ml)
C
(kDa) M 1 Fractions 14
eIF3 subunit:
a-FLAG
c-Strep
b
d-HA
l
e/f/g/h/i-His10/m
k
e/f/h/m
g
i-His10
D
eIF3 native (50 nM)
875 ± 98 kDa
eIF3 rec. (50 nM)
888 ± 86 kDa
Normalised counts
Mass (kDa)
E
eIF3bgi (100 nM)
166 ± 22 kDa
eIF3-Δbgi (50 nM)
525 ± 72 kDa
617 ± 97 kDa
eIF3-Δbgi + eIF3bgi (50 nM each)
202 ± 44 kDa
489 ± 87 kDa
791 ± 114 kDa
Normalised counts
Mass (kDa)
F
eIF3-Δbgi
150 mM KCl
250 mM KCl
400 mM KCl
A280 (mAU)
Elution volume (ml)

Figure S5. Additional analysis of eIF3-∆bgi and eIF3bgi. A. Coomassie-stained SDS polyacrylamide gel showing the insoluble (pellet, P) and soluble (supernatant, SN) fractions of P1 virus generating cells. Unambiguously identified subunits are indicated on the right. B. Chromatograms of analytical SEC runs using a Superose6 3.2/300 column of isolated eIF3-∆bgi, eIF3bgi, a mix of the two subcomplexes at equimolar ratio (eIF3-∆bgi + eIF3bgi), or complete eIF3 obtained by purification of mixed cell pellets. The asterisk marks the exclusion (void) volume. The molecular weights were determined from a run of globular standard proteins. All SEC samples contained 10 µM of the corresponding complexes in 25 µl. C. Coomassie-stained SDS polyacrylamide of the fractions from the four analytic SEC runs as highlighted in (B); gels are colour-coded as curves in (B). D. and E. Mass photometry histograms of eIF3bgi (theor. MW: 166 kDa), eIF3-∆bgi (theor. MW: 609 kDa) native eIF3 (theor. MW: 764 kDa) and recombinant eIF3 (theor. MW: 775 kDa), acquired at the indicated concentrations. Masses of the subcomplexes were derived from a Gaussian fit. For the interaction experiment between eIF3-∆bgi and eIF3bgi, the two subcomplexes were incubated at equimolar ratio at 500 nM for 10 min at 30 °C, then diluted to 50 nM for measurement. F. Chromatogram of analytical SEC runs using a Superose6 3.2/300 column of isolated eIF3-∆bgi at KCl concentrations of 150 mM (same KCl concentration as in (B)), 250 mM or 400 mM respectively. The asterisk marks the exclusion (void) volume. All SEC samples contained 1.5 µM protein in 25 µl.
