# Supplementary material for: Purification and characterization of recombinant human translation initiation factor eIF3
Source: Protein Sci. 2025 Dec 23;35(1):e70388. doi: 10.1002/pro.70388 (PMC12723715; doi:10.1002/pro.70388)
Supplement: Supplementary file 6 — Figure S6. Schematic representation of the eIF2 subunits organization in the plasmids. (a) Arrangement of individual eIF2 subunits in pACEBac plasmids and final combination on the pBIG1A plasmid. (b) Enzymatic digestion of the positive pBIG1A‐eIF2abg clone. PmeI digestion release the entire fragment containing the three subunits (5 Kb approx.) and XbaI + BamHI digestion releases individual subunits (sizes label on the panel). [file PRO-35-e70388-s004.pdf]

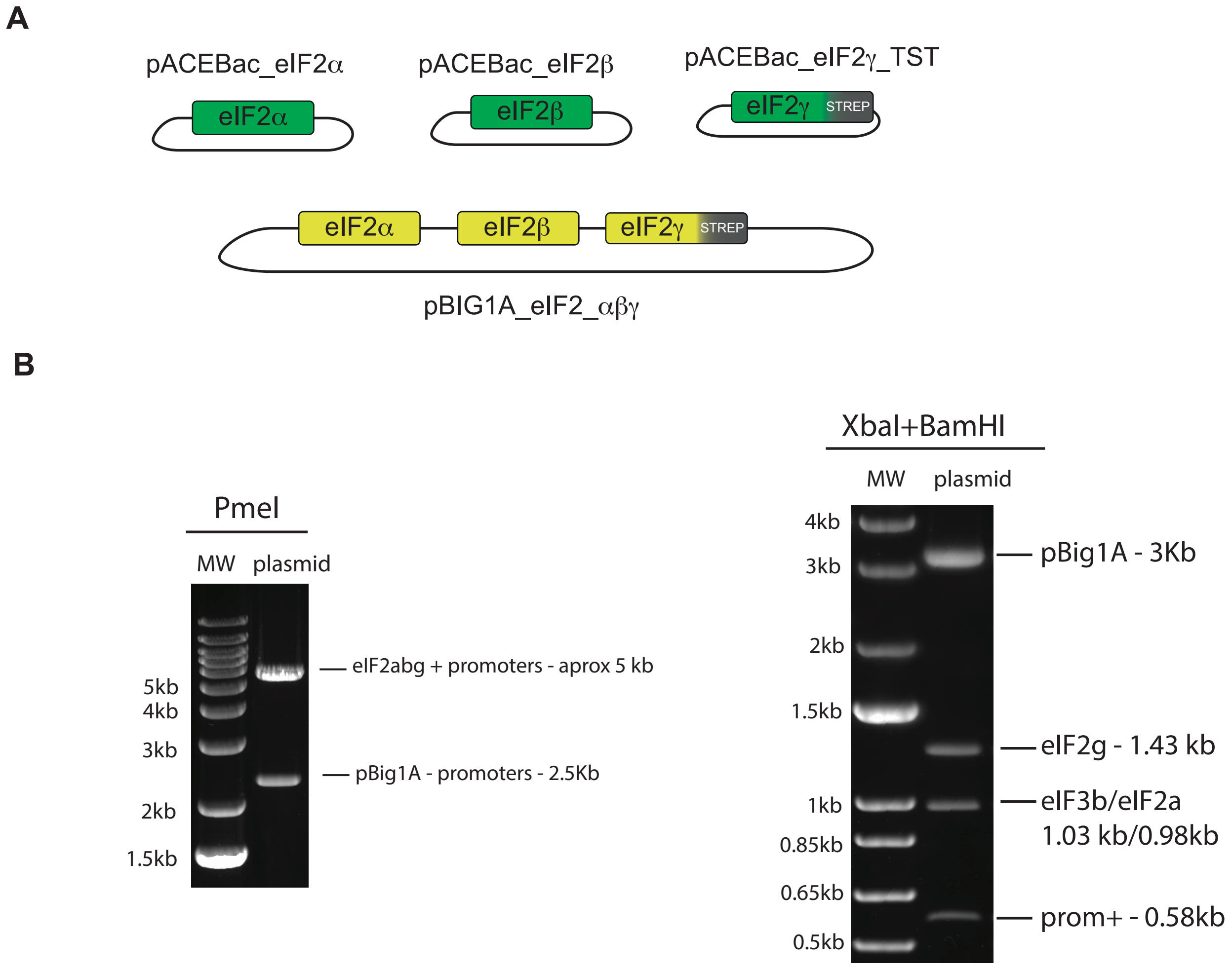

Figure S6. Schematic representation of the eIF2 subunits organization in the plasmids. (A) Arrangement of indiviual eIF2 subunits in pACEBac pplasmids and final combination on the pBIG1A plasmid. (B.) Enzymatic digestion of the positive pBIG1A-eIF2αβγ clone. PmeI digestion release the entire fragment containing the three subunits (5 Kb aprox) and XbaI+BamHI digestion releases individual sububnits (sizes label on the panel).
